# The Role of Lifestyle Modification with Second-Generation Anti-obesity Medications: Comparisons, Questions, and Clinical Opportunities

**DOI:** 10.1007/s13679-023-00534-z

**Published:** 2023-12-02

**Authors:** Thomas A. Wadden, Ariana M. Chao, Molly Moore, Jena S. Tronieri, Adam Gilden, Anastassia Amaro, Sharon Leonard, John M. Jakicic

**Affiliations:** 1 Department of Psychiatry, Perelman School of Medicine at the University of Pennsylvania, 3535 Market Street, Suite 3027, Philadelphia, PA 19104 USA; 2 Johns Hopkins University School of Nursing, Baltimore, MD USA; 3 Department of Medicine, University of Colorado School of Medicine, Denver, CO USA; 4 Department of Medicine, Perelman School of Medicine at the University of Pennsylvania, Philadelphia, PA USA; 5 Department of Medicine, Medical Center, Kansas University, Kansas City, KS USA

**Keywords:** Obesity, Weight loss, Lifestyle intervention, Pharmacotherapy, Body composition, Cardiometabolic health

## Abstract

**Purpose of Review:**

This review examines lifestyle modification for obesity management with the goal of identifying treatment components that could support the use of a new generation of anti-obesity medications (AOMs).

**Recent Findings:**

Semaglutide reliably reduces baseline body weight by approximately 15% at 68 weeks, in contrast to 5–10% for lifestyle modification. Tirzepatide induces mean losses as great as 20.9%. Both medications reduce energy intake by markedly enhancing satiation and decreasing hunger, and they appear to lessen the need for traditional cognitive and behavioral strategies (e.g., monitoring food intake) to achieve calorie restriction. Little, however, is known about whether patients who lose weight with these AOMs adopt healthy diet and activity patterns needed to optimize body composition, cardiometabolic health, and quality of life.

**Summary:**

When used with the new AOMs, the focus of lifestyle modification is likely to change from inducing weight loss (through calorie restriction) to facilitating patients’ adoption of dietary and activity patterns that will promote optimal changes in body composition and overall health.

## Introduction

A new generation of anti-obesity medications (AOMs), inaugurated in 2021 with the US Food and Drug Administration’s (FDA) approval of semaglutide 2.4 mg, holds real promise of transforming the management of obesity [1••]. These novel nutrient stimulated hormone-based therapies reduce baseline body weight by an average of 15% or more [2••, 3•]. The FDA recommends that semaglutide be used as an adjunct to a reduced calorie diet and increased physical activity [4], long considered the cornerstone of obesity management when combined with behavior-change strategies [5, 6]. The efficacy, however, of this medication and of the recently FDA-approved tirzepatide (November 8, 2023) raises questions about the specific diet and activity counseling needed. The intensity of traditional lifestyle modification likely can be reduced, but new diet and activity challenges may arise with the larger weight losses produced by what Garvey [7] has called “second-generation” AOMs. This review examines these and related issues after first summarizing the mechanisms of action and efficacy of both lifestyle modification and two new AOMs.

## Lifestyle Modification for Overweight and Obesity

Lifestyle modification provides behavioral and cognitive strategies to help individuals consciously regulate their energy intake (i.e., food) and expenditure (i.e., physical activity) [8–12]. This approach fortifies the highly integrated gut-to-brain neuroendocrine system, which evolved in an environment of food scarcity (rather than abundance) to facilitate energy homeostasis and prevent the loss of body mass, vital to survival [13]. In the USA and other high-income nations, the neuroendocrine regulation of body weight has been largely overwhelmed for the past 40 years by what Brownell and Horgen have labeled a toxic food environment [14]. It relentlessly markets large portions of cheap, highly processed, and highly palatable foods (e.g., fat, sugar, salt) that excite neural reward pathways that encourage excess eating [14, 15]. This mismatch between our ancient appetite-regulatory system and the current food environment [16], combined with marked reductions in energy expenditure at work and at home [14], explains much of the obesity epidemic.

As described elsewhere, traditional lifestyle modification begins with daily monitoring of food intake and physical activity to educate individuals about energy balance [8–12]. Patients who are new to tracking are often surprised by the high calorie content of morning juices, fast-food lunches, and evening snacks and beverages, as well as by the modest calorie expenditure of a 30-min walk. Individuals are encouraged to reduce portion sizes, as well as excess fat and sugar, to decrease intake by 500–750 kcal/day [6]. This deficit typically can be achieved in persons who weigh < 250 lb by aiming for 1200–1499 kcal/day or 1500–1800 kcal/day if ≥ 250 lb [6, 10]. Consumption of a self-selected diet is recommended with approximately 15% of energy from protein, 20–35% from fat, and the remainder from carbohydrate, although this mix can be adjusted (e.g., 25% protein) to meet dietary preferences [6]. Strategies such as shopping from a list, storing foods out of sight, and preparing more meals at home (i.e., less ordering in and eating out) help distance individuals from the food environment [8–12]. Cognitive techniques facilitate satiation, coping with hunger and cravings, and recovery from dietary lapses. The initial activity goal is walking (or other aerobic activity) for ≥ 150 min/week [6, 17], eventually increasing to ≥ 250 min/week for weight-loss maintenance [18]. Participants are instructed to weigh themselves at least weekly and to use problem solving to adjust their eating and activity in response to their weight change [8–12].

### End-of-Treatment Outcomes

**Fig. 1:**
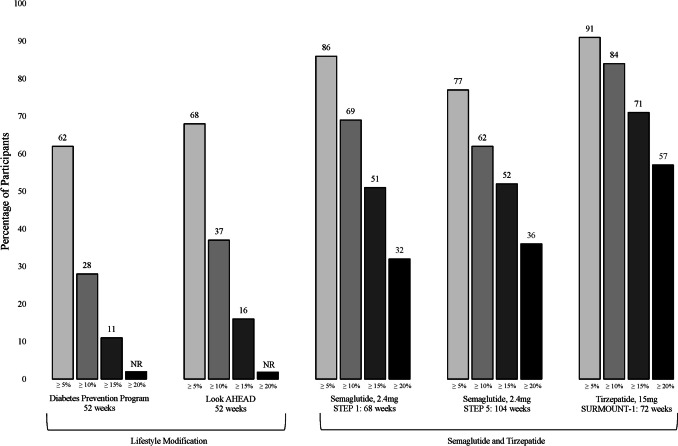
Percentage of participants who achieved categorical losses ≥ 5%, ≥ 10%, ≥ 15%, and ≥ 20% of baseline body weight with intensive lifestyle modification alone as compared with semaglutide and tirzepatide (combined with approximately monthly, brief lifestyle counseling) (NR, not reported)

Individuals must devote substantial time, thought, and planning to cognitive self-regulation [10]. This includes triumphing daily over exhortations (e.g., food advertising) to eat more, as well as increasing physical activity in an environment that implicitly discourages it. These efforts are supported by participating in a structured 1-year program that provides, in the first 6 months, at least 14 individual or group sessions with a trained interventionist [6, 8, 19]. (Treatment is often weekly for the first 3–6 months.) Such regimens typically induce a mean 5–10% reduction in baseline weight at 1 year (compared with 1–2% for controls), as in the Diabetes Prevention Program and Look AHEAD trials [6, 8, 20–22]. Approximately 55–65% of participants lose ≥ 5% of weight, 30–35% lose ≥ 10%, and 10–15% achieve ≥ 15% [23] (Fig. 1). These reductions are associated in a generally linear manner with improvements in cardiovascular disease (CVD) risk factors, quality of life, and prevention of type 2 diabetes [24–26]. Variation of macronutrient composition (e.g., carbohydrate, protein) has little effect on 1- to 2-year weight loss but may improve some health conditions (type 2 diabetes) [27, 28]. Combining lifestyle modification with high-protein, very-low-calorie diets (≤ 800 kcal/day) increases short-term weight loss to 12–20% but with more costs, side effects, and weight regain than less restrictive diets [29].

### Post-Treatment Outcomes and Challenges

Limitations of lifestyle modification include the plateauing of weight loss at 6–9 months [30], even when participants still have obesity and may receive continued counseling [6, 31]. In addition, individuals regain a mean one-third of lost weight in the year following treatment discontinuation, with further regain over time [6, 30]. Monthly or more frequent weight-loss-maintenance therapy delays but does not prevent regain, particularly after treatment concludes [6, 30, 31]. Some individuals, as in the National Weight Control Registry, achieve and maintain losses ≥ 15%, but they work tenaciously [32]. Registry participants report eating 1381 kcal/day and engaging in 60 min/day of physical activity. The high activity level appears necessary to compensate for greater-than-expected reductions in energy expenditure, both at rest and during activity, which accompany weight loss (i.e., metabolic adaptation) [33–35]. A final shortcoming of lifestyle modification is its limited availability outside of academic medical centers (and some commercial and community-based programs), making it virtually impossible for primary care providers to offer all patients with obesity “intensive, multicomponent behavioral interventions,” as recommended by the US Preventive Services Task Force [36]. Digitally delivered interventions have increased the reach of lifestyle modification but with reduced efficacy to date [8].

## Second-Generation AOMs: Mechanisms of Action and Safety Findings

Semaglutide 2.4 mg produces roughly twice the weight loss, on average, as traditional lifestyle modification and also represented a major advancement over the four previously approved medications for chronic weight management—orlistat, naltrexone-bupropion extended release (ER), phentermine-topiramate ER, and liraglutide 3.0 mg [37–39]. The last two medications are the most effective of the four, inducing mean 1-year reductions of 8–11% of baseline weight when combined with approximately monthly lifestyle counseling [37–39]. AOMs are considered potentially appropriate for individuals with a BMI ≥ 30 kg/m^2^ or a BMI ≥ 27 kg/m^2^ with an obesity-related comorbidity (e.g., hypertension). (We note that in 2020, the US FDA and other regulatory bodies approved setmelanotide for chronic treatment in adults and children at least 6 years of age with rare monogenic or syndromic obesities [40].)

Liraglutide 3.0 mg and semaglutide 2.4 mg are both glucagon-like peptide 1 (GLP-1) receptor agonists [41]. GLP-1 is a native hormone that is released from L-cells of the small intestine and colon in response to nutrient (meal) intake. It binds to GLP-1 receptors expressed in pancreatic beta cells and the heart, as well as in appetite-regulating centers in the hindbrain, hypothalamus, and mesolimbic pathway [41, 42]. GLP-1 stimulates insulin secretion and inhibits glucagon release (in a glucose-dependent manner); it reduces energy intake by slowing gastric emptying and enhancing satiety signaling [41]. The half-life of native GLP-1 is approximately 2–3 min because of degradation by dipeptidyl peptidase 4 (DPP-4). Synthetic GLP-1 receptor agonists (GLP-1RA) are designed to resist this degradation. Liraglutide 3.0 mg has a half-life of approximately 13 h, thus, requiring daily subcutaneous dosing [41].

In contrast, semaglutide 2.4 mg has a half-life of about 180 h, which allows once-weekly subcutaneous dosing [1••, 3•]. This supraphysiologic dose of GLP-1 is associated with robust improvements in objective and subjective appetite control, in the absence of lifestyle counseling. At 20 weeks, semaglutide 2.4 mg, relative to placebo, reduced ad libitum energy intake by 35% during a laboratory lunch test meal. The mean reduction in energy intake, from baseline, was 377 kcal for semaglutide versus 152 kcal for placebo [42]. Semaglutide, relative to placebo, also resulted in larger self-reported reductions in hunger and food cravings, increased fullness and satiety, better control of eating, and lower preference for energy-dense foods [43].

Tirzepatide combines—in a single molecule—glucose-dependent insulinotropic polypeptide (GIP) and GLP-1 receptor agonism [2••]. At the time of this writing, the medication is approved in the US and European Union as a once-weekly subcutaneous injection for type 2 diabetes and, as noted, was recently approved in the US for overweight/obesity [3•]. The mechanisms by which tirzepatide’s dual receptor agonism increases weight loss, relative to GLP-1 agonism alone, are incompletely understood [44]. In adults with type 2 diabetes, tirzepatide 15 mg once weekly reduced energy intake, during an ad libitum lunch, from baseline to week 28, by 348 kcal compared to 39 kcal for placebo [45]. The reduction in energy intake with tirzepatide did not differ significantly from that in participants who received semaglutide 1 mg (with a reduction of 284 kcal). Tirzepatide was associated with significant improvements in self-reported appetite control compared with placebo [45]. By consistently reducing hunger (which initiates eating), increasing satiation and satiety (which terminate meal intake and extend the inter-meal interval, respectively), and potentially decreasing hedonic eating [45–47], the authors view the new-generation AOMs as reducing patients’ responsiveness—or vulnerability—to the toxic food environment.

We note that numerous other medications, with more complex mechanisms of action, are currently being developed [3•]. These include retatrutide, a triple-hormone-receptor agonist (GIP/GLP-1/Glucagon) [48], which was shown in a phase-2 study to reduce baseline weight by as much as 17.5% at 24 weeks and 24.2% at 48 weeks. Combining semaglutide 2.4 mg with an amylin-receptor agonist, cagrilintide 2.4 mg (i.e., CagriSema 2.4 mg), was similarly shown in a phase-1b trial to decrease baseline weight by 17.1% at 20 weeks, with substantial additional weight loss projected with continued use [49]. These and other medications are not discussed further due to space limitations and because their consideration for approval is more than a year away.

### Safety

Across the phase 3 trials of semaglutide and tirzepatide (the latter still being completed), the most common adverse events were gastrointestinal in nature, including nausea, diarrhea, vomiting, constipation, dyspepsia, and abdominal pain [1••, 2••, 50, 51•, 52••, 53••, 54–57]. These symptoms were typically mild to moderate in severity. The rate of discontinuation due to adverse events was approximately 6–7% in participants treated by semaglutide 2.4 mg [1••] or by tirzepatide 10–15 mg [2••] vs 2.5–3% for placebo-treated participants. The rate of serious adverse events (SAEs) with semaglutide was approximately 7.7–9.8% vs 5.6–6.4% for placebo [1••], with similar rates for tirzepatide vs placebo [2••]. Adverse events of special interest included gallbladder-related disorders, acute pancreatitis, acute renal insufficiency, hypoglycemia, and injection site reactions [1••, 2••].

Semaglutide and tirzepatide are both titrated upward over 16 to 20 weeks to limit GI side effects described previously [1••, 2••]. Semaglutide, for example, is introduced at 0.25 mg for 4 weeks and then increased at 4-week intervals to 0.5, 1.0, 1.7, and 2.4 mg. In phase 3 trials, lower doses were allowed for maintenance if intolerable side effects occurred. In clinical practice, the dose may be increased even more slowly, according to patient preference, side effects, and progression of weight loss.

## Efficacy of Semaglutide 2.4 mg for Overweight/Obesity

To date, the efficacy of semaglutide 2.4 mg has been evaluated in a series of seven trials called semaglutide treatment effect in people with obesity (STEP). (Results of an eighth trial have yet to be published.) Participants in all trials but STEP 3 (described later) received brief (15 min) lifestyle counseling visits approximately every 4 weeks to help them meet the study goals of achieving a 500-kcal/day deficit and ≥ 150 min/week of moderate to vigorous physical activity (principally walking). Counseling was delivered by registered dietitians (RD) or other qualified professionals who were free to advise participants as they saw fit. In some studies, the sponsor provided supplementary materials (e.g., food plate, toolbox) to be shared with participants at the RD’s discretion.

### End-of-Treatment Outcomes in Selected Trials

In STEP 1, participants with overweight/obesity (but not type 2 diabetes) treated with semaglutide lost a mean 14.9% of baseline weight at 68 weeks, compared with 2.4% for placebo (both combined with 17 brief lifestyle visits) [1••]. Roughly 75% of semaglutide-treated participants lost ≥ 10% of weight, 50% lost ≥ 15%, and one-third reduced by ≥ 20% (Fig. 1). The approximately 15% loss in STEP 1 was replicated in five additional STEP trials that included similar participants but addressed other study questions [51•, 52••, 53••, 54, 55, 57] (Table 1). In STEP 2, participants with type 2 diabetes (and overweight/obesity) lost only 9.6% with semaglutide 2.4 mg [50]. Other AOMs have yielded comparably smaller weight losses in patients with versus without type 2 diabetes [58].

**Table 1:** Summary of end-of-treatment changes in key outcomes in seven STEP trials (semaglutide) and three SURMOUNT trials (tirzepatide)

Trial name	Treatment arms (N); trial duration, weeks	Baseline weight, % Δ	SBP, mm Hg	DBP, mm Hg	HbA1c, percentage points	Triglycerides^a^	Total cholesterol^a^	HDL^a^	LDL^a^	C-reactive protein^a^	Waist circumference, cm	SF-36, physical function score
STEP 1	Semaglutide 2.4 mg + lifestyle intervention (1306); 68 wk	− 14.9*	− 6.2*	− 2.8*	− 0.5*	0.78*	0.97*	1.05*	0.97*	0.47*	− 13.5*	2.2*
	Placebo + lifestyle intervention (655)	− 2.4	− 1.1	− 0.4	− 0.2	0.93	1.00	1.01	1.01	0.85	− 4.1	0.4
STEP 2	Semaglutide 2.4 mg + lifestyle intervention (404); 68 wk	− 9.6*	− 3.9*	− 1.6	− 1.6*	0.78	0.99	1.07	1.00	0.51	− 9.4*	2.5*
	Semaglutide 1.0 mg + lifestyle intervention (403)^	− 7.0	− 2.9	− 0.6	− 1.5	0.83	0.98	1.05	0.99	0.58	− 6.7	2.4
	Placebo + lifestyle intervention (403)	− 3.4	− 0.5	− 0.9	− 0.4	0.91	0.99	1.04	1.00	0.83	− 4.5	1.0
STEP 3	Semaglutide 2.4 mg + low calorie diet for the first 8 weeks + intensive behavioral therapy (407); 68 wk	− 16.0*	− 5.6*	− 3.0*	− 0.5*	− 22.50%*	− 3.80%*	6.50%	− 4.70%*	59.60%*	− 14.6*	2.4
	Placebo + low calorie diet for the first 8 weeks + intensive behavioral therapy (204)	− 5.7	− 1.6	− 0.8	− 0.3	− 6.50%	2.10%	5.00%	2.60%	− 22.90%	− 6.3	1.6
STEP 4^b^	Continued semaglutide 2.4 mg + lifestyle intervention (535); 68 wk	− 7.9*	0.5*	0.3	− 0.1*	− 6.00%*	5.00%*	18.00%	1.00%*	NR	− 6.4*	1.0*
	Switched to placebo + lifestyle intervention (268)	6.9	4.4	0.9	0.1	15.00%	11.00%	18.00%	8.00%	NR	3.3	− 1.5
STEP 5	Semaglutide 2.4 mg + lifestyle intervention (152); 104 wk	− 15.2*	− 5.7*	− 4.4	− 0.4	− 19.00%	− 3.30%	9.60%	− 6.10%	− 56.70%	− 14.4*	NR
	Placebo + lifestyle intervention (152)	− 2.6	− 1.6	− 0.8	− 0.1	3.70%	1.40%	8.10%	− 2.70%	− 7.80%	− 5.2	NR
STEP 6	Semaglutide 2.4 mg + lifestyle intervention (199); 68 wk	− 13.2*	− 10.8	− 5.3	− 0.9	0.79	0.91	1.09	0.85	0.42	− 11.1*	0.8
	Semaglutide 1.7 mg + lifestyle intervention (101)	− 9.6*	− 10.8	− 4.6	− 0.9	0.80	0.93	1.07	0.90	0.62	− 7.7*	− 0.1
	Placebo + lifestyle intervention (101)	− 2.1	− 5.3	− 2.2	− 0.0	1.06	1.01	1.06	0.96	0.89	− 1.8	− 0.3
STEP 8	Semaglutide 2.4 mg + lifestyle intervention (126); 68 wk	− 15.8*	− 5.7	− 5.0	− 0.2	− 20.70%	− 7.10%	− 0.30%	− 6.50%	− 52.60%	− 13.2	NR
	Liraglutide 3.0 mg + lifestyle intervention (127)	− 6.4*	− 2.9	− 0.5	− 0.1	− 11.00%	− 0.10%	1.90%	0.90%	− 24.50%	− 6.6	NR
	Placebo + lifestyle intervention (85)	− 1.9	3.2	0.7	0.1	− 3.20%	− 3.30%	− 0.90%	− 1.10%	− 20.10%	− 2.0	NR
SURMOUNT 1^c^	Tirzepatide 5.0 mg + lifestyle intervention (630); 72 wk	− 15.0*	− 7.0	− 5.2	− 0.4	− 24.30%	− 4.90%	7.00%	− 5.30%	NR	− 14.0*	3.9
	Tirzepatide 10.0 mg + lifestyle intervention (636)	− 19.5*	− 8.2	− 5.5	− 0.5	− 27.00%	− 5.60%	8.60%	− 6.60%	NR	− 17.7*	3.9
	Tirzepatide 15.0 mg + lifestyle intervention (630)	− 20.9*	− 7.6	− 4.6	− 0.5	− 31.40%	− 7.40%	8.20%	− 8.60%	NR	− 18.5*	4.2
	Placebo + lifestyle intervention (643)	− 3.1	− 1.2	− 1.0	− 0.1	− 6.30%	− 1.10%	0.20%	− 0.90%	NR	− 4.0	1.9
SURMOUNT 2^d^	Tirzepatide 10.0 mg + lifestyle intervention (312); 72 wk	− 12.8*	− 5.9	− 2.1	− 2.1*	− 26.80%*	− 3.00%*	6.90%*	2.30%	NR	− 10.8*	3.4*
	Tirzepatide 15.0 mg + lifestyle intervention (311)	− 14.7*	− 7.7	− 2.9	− 2.1*	− 30.60%*	− 2.20%*	9.60%*	3.20%	NR	− 13.1*	3.8*
	Placebo + lifestyle intervention (315)	− 3.2	− 1.2	− 0.3	− 0.5	− 5.80%	2.10%	1.10%	6.30%	NR	− 3.3	1.6
SURMOUNT 3^c,e^	Tirzepatide MTD + quarterly lifestyle intervention (287); 72 wk	− 18.4*	− 5.1	− 3.2	− 0.5	− 25.80%	− 3.00%	15.40%	− 6.10%	NR	− 14.6*	3.3
	Placebo + quarterly lifestyle intervention (292)	2.5	4.1	2.3	0.0	3.00%	5.20%	3.60%	6.10%	NR	0.2	− 0.6

This table is adapted and updated from Table 1 in reference [57], originally published in Trends in Cardiovascular Medicine, vol 33: Chao, AM, Tronieri JS, Amaro A, Wadden TA; Semaglutide for the treatment of obesity, pp 159–166, Copyright Elsevier 2023

*SBP* systolic blood pressure, *DBP* diastolic blood pressure, *NR* not reported, *MTD* max tolerated dose

^*^p < 0.05 with comparison to placebo. ^Formal statistical comparisons were not performed between semaglutide 1.0 mg and placebo

^a^For STEP trials 1, 2, and 6, lipid values presented are a ratio of week 68 to baseline. For STEP trials 3, 4, 5, 8, and SURMOUNT trials 1, 2, and 3, values presented are percent change

^b^All participants were treated with semaglutide from baseline to 20 weeks. Results presented are changes from weeks 20–68

^c^Efficacy estimand used for SBP, DBP, HbA1c, triglycerides, total cholesterol, HDL, LDL, and SF-36 physical functioning score. Formal statistical comparisons were not performed

^d^Efficacy estimand used for SBP, DBP, triglycerides, total cholesterol, HDL, LDL, and SF-36 physical functioning score

^e^Prior to randomization, all participants were required to complete a 12-week lead-in program that provided intensive lifestyle intervention alone. Those who successfully lost ≥ 5% of their baseline weight (and met other eligibility criteria) were randomly assigned to tirzepatide or placebo. Results shown in the table are from randomization to week 72 and do not reflect changes in weight (− 6.9% reduction in lead-in weight during the 12 weeks) and other outcomes that occurred during the lead-in program

Weight loss with semaglutide was associated with clinically meaningful improvements in several cardiometabolic risk factors (e.g., blood pressure and triglyceride levels) and in self-reported physical function (Table 1). On November 11, 2023, Lincoff et al. reported that semaglutide 2.4 mg reduced the risk of major adverse cardiovascular events (MACE) by 20%, compared with placebo, in persons with overweight/obesity and a history of CVD (but not type 2 diabetes) [59]. This is the first such finding from a randomized controlled trial of an obesity treatment. The results are consistent with those for both liraglutide 1.8 mg and semaglutide 1.0 mg in persons with type 2 diabetes and CVD (or a high risk of it) [60]. The previously discussed Look AHEAD study did not reduce MACE in patients with type 2 diabetes and overweight/obesity, potentially because of insufficient weight loss [61].

### Comparative Treatment Outcomes

Two STEP trials compared the efficacy of semaglutide with either another FDA-approved AOM or with intensive lifestyle modification. STEP 8 observed mean 68-week reductions in baseline weight of 15.8% with semaglutide versus 6.4% with liraglutide 3.0 mg (and 1.9% for pooled placebo) [55]. Semaglutide has not been compared directly with the older orlistat, naltrexone-bupropion, or phentermine-topiramate.

STEP 3 sought to maximize weight loss with semaglutide by combining it with intensive lifestyle modification [51•]. This approach was based on prior evidence of additive weight loss with combined behavioral and pharmacologic therapies [62]. All participants in STEP 3 received 30 brief visits with an RD over 68 weeks, which included, for the first 8 weeks, a 1000–1200-kcal/day meal-replacement diet, given the greater weight loss with this approach compared with an isocaloric, self-selected diet [11]. Participants who received this program, with placebo, lost approximately 8% of baseline weight at week 28, which declined to 5.7% at week 68, likely because of decreased counseling visits in later months. Participants assigned to semaglutide, with the same lifestyle intervention, lost 16.0% of baseline weight at trial completion and achieved significantly greater improvements than placebo-treated participants on multiple measures of cardiometabolic risk, as expected with greater weight loss (Table 1).

Extrapolating across STEP 1 and 3—with needed caution—the addition of intensive lifestyle modification and meal replacements (MRs) in STEP 3 appeared to increase early weight reduction with semaglutide but produced only marginally greater end-of-treatment weight loss (1.1 percentage points) than in STEP 1, in which semaglutide was delivered with less intensive counseling (17 visits and no MRs). The anticipated additive weight loss was not observed. Instead, the medication appeared to help individuals achieve the same long-term weight loss, regardless of the intensity of the initial lifestyle counseling. The two studies, taken together, also suggest that semaglutide (with approximately monthly lifestyle counseling) is approximately twice as effective as high-intensity lifestyle counseling alone (as provided in STEP 3), although a randomized controlled trial is needed to confirm this hypothesis and to compare the cost-effectiveness of the two approaches.

As discussed later, the optimal content and frequency of lifestyle counseling with semaglutide require further investigation. The medication’s efficacy in improving hunger, satiation, and other aspects of appetite control appears to decrease the short-term need for traditional behavioral strategies to achieve calorie restriction. The authors have heard multiple patients’ remark, “I’m just not thinking as much about food and eating,” a welcomed reduction in food preoccupation (“food noise”) and the usual cognitive demands of weight management. Semaglutide, a biological therapy, appears to modify eating behavior with greater ease and efficiency than traditional behavioral therapy. As a colleague recently noted, “These medications make lifestyle modification ‘a smaller ask’ for both patients and practitioners.”

### Post-treatment Outcomes and Challenges

Individuals regain weight quickly after discontinuing semaglutide. A subset of 228 STEP-1 participants, who lost a mean 17.3% of baseline weight at week 68, regained an average of two-thirds of their loss (11.6 percentage points) 1 year after discontinuing medication [63••]. (The placebo group regained 1.9 percentage points of a 2.0% loss.) Previously improved cardiometabolic outcomes also reverted to baseline. These results mirror those from STEP 4 in which semaglutide-treated participants lost a mean 10.6% of baseline (run-in) weight in 20 weeks but, when randomly switched to placebo, regained 4.9% percentage points (about half) in the ensuing 48 weeks [52••]. In contrast, participants randomly assigned to remain on semaglutide lost an additional 6.8% of run-in weight. These two trials confirm the chronicity of obesity and the clear need for its long-term (indefinite) treatment, as with other chronic diseases [53••, 64]. STEP 5 illustrated the benefit of such care. Semaglutide-treated participants lost approximately 15% of baseline weight at 52 weeks and maintained this full loss at 104 weeks, while still receiving medication, and had expected improvements in cardiometabolic risk factors and quality of life [53••] (Table 1).

Despite its apparent superiority to lifestyle modification, semaglutide (as well as tirzepatide) shares challenges with the former approach, including that a majority of patients continue to have obesity after treatment. Participants’ mean BMI in STEP 5 declined from 38.6 to 32.8 kg/m^2^, understandably hastening the search for combination therapies to induce larger losses [3, 49]. A similar challenge concerns access to treatment, which is currently imperiled by semaglutide’s variable insurance coverage, for obesity, and by very high out-of-pocket costs in many countries (about $1300/month in the USA). These issues must be resolved to make chronic obesity management a reality. Once addressed, patients—and their practitioners—must embrace long-term adherence to second-generation AOMs, particularly in view of data showing discontinuation of earlier, less expensive medications after only 2 to 4 months [65].

## Efficacy of Tirzepatide for Overweight and Obesity

The safety and efficacy of once-weekly tirzepatide for overweight and obesity are being evaluated in the SURMOUNT trials, with the results of three studies published at the time of this writing [2••, 56, 66].

### End-of-Treatment Outcomes in Selected Trials

In the 72-week SURMOUNT-1 study, participants with overweight/obesity (but not diabetes) lost a mean 15.0% of baseline weight with tirzepatide 5 mg, 19.5% with 10 mg, and 20.9% with 15 mg, compared to 3.1% for placebo [2••]. (All participants received lifestyle counseling which included prescription of a 500-kcal/day deficit and ≥ 150 min/week of physical activity.) More than 75% of participants on the 10 and 15 mg doses lost ≥ 10% of baseline weight, more than 65% lost ≥ 15%, and fully 50% lost ≥ 20% (Fig. 1). In addition, 32.3% and 36.2% of these participants, respectively, lost ≥ 25% of baseline weight (not shown in Fig. 1). Tirzepatide-treated participants achieved significantly greater improvements in cardiometabolic risk factors and physical function than those on placebo.

In SURMOUNT-2, after 72 weeks of intervention, participants with type 2 diabetes and overweight/obesity lost 12.8% of baseline weight on tirzepatide 10 mg and 14.7% on tirzepatide 15 mg, compared to 3.2% for placebo [56]. These participants lost approximately five percentage points less body weight than those in SURMOUNT-1, who were free of type 2 diabetes. This finding parallels that for semaglutide, as discussed earlier. Data from these two SURMOUNT trials suggest that tirzepatide 10–15 mg produces an approximately five percentage point greater reduction in baseline weight than semaglutide 2.4 mg, a hypothesis that is currently being tested in a randomized controlled trial.

SURMOUNT-3 examined the effects of administering tirzepatide 10–15 mg/day after participants were first required to lose at least 5% of their initial body weight during a 12-week lead-in program that provided intensive lifestyle intervention [66]. The intervention included eight visits with an RD, who prescribed a 1200–1500-kcal/day diet, with up to two meal replacements/day, as well as 150 min/week of physical activity. The 579 participants who met the ≥ 5% weight loss criterion, and were otherwise eligible to continue in the study, lost a mean 6.9% of baseline weight during the 12-week program and were randomly assigned to tirzepatide (maximally tolerated dose of 10–15 mg) or placebo for 72 weeks. Tirzepatide-treated participants lost an additional 18.4% of weight from randomization to week 72, compared with a gain of 2.5% for those assigned placebo. (All participants received only quarterly RD visits during the randomized trial.) Approximately 65% of participants on tirzepatide, compared with 4.2% on placebo, lost 15% or more of their randomization weight. The additional weight reduction with tirzepatide was associated with further clinically meaningful improvement in several health outcomes (e.g., blood pressure, waist circumference, lipids, HbA1c, and physical function), which were beyond those achieved in the lead-in period and which were in sharp contrast to the deterioration in these measures in the placebo group. Tirzepatide also facilitated the maintenance of the original lead-in weight loss (of 6.9%): 94.0% of tirzepatide-treated participants compared to 43.8% of placebo maintained 80% or more of their initial 12-week loss. As measured from the start of the lead-in program to the end of the randomized trial—a total of 84 weeks—participants assigned to tirzepatide achieved a cumulative 24.3% reduction in baseline body weight, compared with 4.5% for those on placebo.

These findings suggest a useful treatment option for patients who, following successful weight loss with intensive lifestyle modification (e.g., 7–10%), need to reduce further to achieve optimal control of an obesity-related complication, such as obstructive sleep apnea. The new AOMs also could benefit “do-it-yourself losers” whose weight reduction plateaus after a 5% loss, despite their continued efforts and desire to lose more. Tirzepatide and semaglutide could be particularly beneficial with patients who are not responsive to intensive lifestyle modification, defined as losing < 2% of baseline weight after five weekly sessions with a trained interventionist [67]. Eligibility to participate in phase 3 trials of both medications required a “history of at least one self-reported unsuccessful dietary effort to lose weight.” This criterion, however, could be interpreted as a failure to lose weight and keep it off, versus not being able to lose weight initially. Controlled trials are needed to assess the benefits of second-generation AOMs in patients who are determined prospectively not to respond to an intensive lifestyle intervention.

Findings from SURMOUNT-3 also suggest that intensive lifestyle intervention and AOMs have additive weight-loss benefit when used sequentially, rather than concurrently, as they were in STEP 3. The additional 18.4% reduction in randomization weight, achieved with tirzepatide after the lead-in period, was only marginally smaller than the mean losses of 19.5% and 20.9% achieved in SURMOUNT-1 with tirzepatide 10 and 15 mg/day, respectively. Sequential, additive weight loss was similarly observed when participants who had lost 6.0% of baseline weight in a low-calorie diet run-in program were randomly assigned to liraglutide 3.0 mg or placebo. Additional weight loss with liraglutide, however, was only one-third of that observed with tirzepatide [68]. The effect of reversing the treatment sequencing, by introducing AOMs first, followed by intensive lifestyle intervention, has not been assessed to date, as discussed later.

Additional SURMOUNT trials are evaluating tirzepatide for inducing additional weight loss with medication continuation vs discontinuation after initial weight reduction with tirzepatide (SURMOUNT-4) and reducing MACE in patients with established CVD and overweight/obesity (SURMOUNT-MMO). Other studies are examining obstructive sleep apnea (SURMOUNT-OSA) and heart failure with preserved ejection fraction (SUMMIT).

### Post-treatment Outcomes and Challenges

We anticipate that post-treatment results and challenges with tirzepatide will be generally similar to those discussed with semaglutide. Further evaluation will follow when the full set of SURMOUNT trials has been completed.

## Knowledge Gaps with Semaglutide and Tirzepatide

The exemplary phase 3 trials of semaglutide and tirzepatide clearly reveal the robust weight losses and improvements in cardiometabolic risk factors produced by both medications. However, important questions remain concerning changes in dietary intake, physical activity, psychosocial function, and related outcomes that may accompany weight reduction with these medications. Answers to these questions should facilitate the development of specific guidelines for lifestyle counseling with the new AOMs. We believe that further study and guidance are perhaps most needed concerning changes in body composition.

### Body Composition

Weight loss with semaglutide and tirzepatide is accompanied by favorable reductions in body fat [1••, 2••]. However, it is also accompanied by reduced lean body mass [1••, 2••, 45, 69, 70], which may influence factors that contribute to body weight regulation and other health outcomes. Lean body mass (LBM) is considered a significant driver of metabolic rate, with a reduction in lean tissue partially contributing to reduced daily energy expenditure [34]. The reduction in LBM may also influence the tonic drive to eat, which may further contribute to influences on body weight regulation [71]. Reduced LBM may be of additional clinical importance given its association with decreased bone mineral density and increased risk of fractures, as well as its relation to metabolic function (e.g., insulin sensitivity) and aerobic capacity [72, 73]. Adults 65 years and older are at increased risk of sarcopenia, characterized by an age-related decrease in skeletal muscle mass, with accompanying losses of strength and physical function [74, 75].

With intensive lifestyle interventions, approximately 15 to 25% of total weight loss is derived from LBM [76, 77]; this amount typically increases with greater energy restriction, as with very-low-calorie diets [77]. With the larger weight losses produced by Roux-en-Y gastric bypass, a systematic review found that the median reduction of lean mass was 31% of total weight [77]. Analyses of subsets of semaglutide- and tirzepatide-treated participants who completed assessments by dual-energy X-ray absorptiometry (DXA) did not include estimates of the percentage of weight loss from LBM. However, supplementary data published with STEP 1 reported that participants lost 10.40 kg of fat mass and 6.92 kg of LBM, suggesting that roughly 40% of total weight loss was derived from lean mass [1••]. This is a high value, which requires verification by a thorough statistical analysis and by checking the assessment methods used, given that placebo-treated participants lost 1.17 kg of fat and an unexpected 1.48 kg of LBM (i.e., roughly 56% loss from lean mass). Data from SURMOUNT-1, presented by Kushner et al. at the 2022 meeting of The Obesity Society, suggested that approximately 25% of weight loss with tirzepatide was derived from LBM and that there were no significant differences in this outcome according to age category [78]. In adults < 50, 50 to < 65, and ≥ 65 years of age, the loss of lean mass was estimated to comprise 27%, 25%, and 24% of total weight loss, respectively. These estimated values also require additional statistical analysis, and the findings should be interpreted with caution given the small number of older participants.

We acknowledge that interpretation of changes in body composition is challenging, particularly since weight gain and obesity are associated with increases in both fat mass and LBM; thus, some loss of LBM is to be expected with weight reduction [79]. In addition, reduced lean mass with weight loss could result primarily from a reduction in low-density/low-quality muscle compared to normal density muscle [80], a finding which could allay concerns. It also may be possible to combine semaglutide and tirzepatide with novel drugs such as bimagrumab, which appears to increase lean mass while reducing fat mass [81]. Large-scale, in-depth investigations of changes in body composition with second-generation AOMs should be a top priority, given the range and vast numbers of patients who are likely to be treated with these drugs in the coming years. Studies should seek to identify risk factors for the excessive loss of lean mass, including age and gender, as well as the rapidity and total amount of body weight lost.

## Dietary Intake with Second-Generation AOMs

The large weight losses achieved in the STEP and SURMOUNT trials suggest that participants were largely successful in achieving the 500-kcal/day energy restriction prescribed. Data from the ad libitum buffet lunch meals, described earlier, provide further evidence of the medications’ efficacy in reducing energy intake [43, 45]. Yet, no results have been published to date concerning participants’ baseline dietary intake, including macronutrient distribution, and how it may have changed with weight loss facilitated by the medications. Similarly, although participants in some trials were instructed to monitor their food intake, findings have not been reported from these data concerning potential changes in the number of daily meals and snacks consumed, the timing of meals, or potential changes in problem eating [82]. We similarly do not know whether greater record keeping was associated with greater weight loss, as found in a trial of liraglutide [83]. We hope that such data will be published over time or, if not available, that additional studies will be conducted to reveal how patients change their food intake to lose weight. What, for example, is the relative importance for weight loss—and for improved cardiometabolic health—of participants’ decreasing their customary portion sizes, while potentially eating the same diet of energy-dense, highly processed foods, versus shifting their intake towards servings of lean protein, whole grains, and more fruits and vegetables? Do semaglutide and tirzepatide facilitate patients not only reducing their energy intake but also adopting a health-promoting dietary pattern that will improve specific cardiometabolic risk factors (e.g., elevated lipids levels) beyond the improvements conferred by weight loss alone? Studies that address these questions will also provide a better understanding of the optimal frequency and focus of lifestyle counseling required with the new AOMs. The recommendations that follow here—and in the next sections—are based on existing guidelines and our observations from clinical practice but await refinement with further research.

### Diet Quality

Since second-generation AOMs substantially decrease the quantity of food consumed, it is important to counsel patients to increase the quality of the foods they eat. Brief dietary assessment can be conducted in clinical settings using 24-h recalls, dietary screeners such as the Rapid Eating and Activity Assessment for Participants (short version) [84], or food frequency questionnaires (Table 2). Patients can also monitor their dietary intake using a paper or electronic diary (e.g., MyFitnessPal) and review a few days’ records with their health care professional or an RD.

**Table 2:** Lifestyle modification for weight management with second-generation anti-obesity medications (AOMs)

	Critical questions	Assessment	Intervention
Dietary intake	During weight loss with AOMs, do patients • Consume an appropriate dietary pattern (including lean proteins, fruits, and vegetables) to promote cardiometabolic health? • Consume adequate amounts of protein to preserve lean body mass? • Maintain adequate hydration? • Limit problem foods to mitigate potential gastrointestinal (GI) side effects related to AOMs?	• Before beginning AOMs, use brief interview or screening questionnaires [84] to assess dietary intake. Repeat this assessment periodically (e.g., every 3 months) • Assess patient’s daily schedule of meals and snacks • Have patients periodically monitor food intake for 2–3 days, particularly for protein and fluids • Evaluate relationship of GI side effects to dietary intake or timing of intake (e.g., high-fat or high-sugar foods, eating late at night)	• Recommend reduced-calorie diet (500 kcal/d deficit) that promotes cardiometabolic health and encourages consumption of lean proteins, fruits, and vegetables and fewer highly processed foods (i.e., high in fat and sugar) • Recommend daily protein intake of at least 0.8 g/kg of body weight, with potentially higher amounts for patients with BMIs ≥ 40 kg/m^2^ • Recommend water and non-caloric fluids to maintain hydration (2.2–3 L/d) • Refer to registered dietitian (RD) for patients with clinically significant problems or who desire more education and support
Physical activity and body composition	During weight loss with AOMs, do patients • Increase their physical activity (planned and lifestyle) to improve their cardiometabolic health (e.g., cardiorespiratory fitness)? • Benefit from strength training (ST)? • Maintain appropriate nutrition and hydration to support increased physical activity?	• Use brief interview, activity diary, or step counter to assess baseline physical activity and changes during treatment • Assess appropriateness of ST based on general health, and potentially with DXA in older adults when body composition, bone mass, or strength are of concern (e.g., sarcopenia) • Assess dietary intake using methods described	• Recommend ≥ 150 min/wk of physical activity (e.g., walking) during weight loss, potentially increasing with weight-loss maintenance • Recommend ST (2 day/wk) as part of general activity program, as appropriate • Refer to certified clinical exercise physiologist for patents with clinically significant problems or who want more education/support
Psychosocial and behavioral issues	During weight loss with AOMs, do patients • Have appropriate mental health and realistic goals for improving weight, health, and quality of life (QOL)? • Based on their psychiatric history, have a risk of psychosocial distress (e.g., suicidal thoughts/behaviors, anxiety, depression, relationship changes) or a desire to continue losing weight below an appropriate BMI (e.g., 20-22 kg/m^2^)? • Need to frequently monitor their food intake and physical activity or to have more than approximately monthly lifestyle contacts • Wish to stop taking AOMs for reasons other than side effects or financial costs (e.g., belief that the medication is no longer working)? • Have plans and strategies for maintaining their weight loss after discontinuing medication?	• Assess mood and psychosocial status at baseline and follow-up visits. Use PHQ-9 [104] and C-SSRS [105] as needed • With weight loss, periodically inquire about changes in mood, sleep, and energy level, as well as satisfaction with work, social interactions, and feelings about themselves (body image). Use PHQ-9 when concerned • Have patients monitor food intake (e.g., MyFitnessPal) and physical activity (e.g., step counter) frequently during first few weeks to support self-learning and behavior change • Discuss patients’ reasons for wishing to stop AOMs and the possible consequences of doing so (e.g., return of appetite and weight) • Assess patients’ strategies for maintaining weight loss without support of AOMs	• With patients who report concerning changes in mood and psychosocial function, or desire to reduce below an appropriate BMI, provide reassurance and education. In clinically significant cases, not responsive to education, refer to a mental health professional. Consider medication dose reduction in patients who have achieved an appropriate BMI (e.g., 20–22 kg/m^2^) • Adjust patients’ frequency of self-monitoring (and lifestyle contacts) accordingly to achieve their goals for eating a healthier diet, increasing physical activity, and other outcomes • Review patients’ potential concerns that “the medication is no longer working.” Provide reassurance and education about the benefits of continued medication use and likely results of discontinuation • With patients’ terminating medication, consider down-titrating the dose over several months to reduce potential rebound in appetite and eating. Assist patients in joining (or developing) a behavioral weight loss maintenance plan, with high levels of physical activity and frequent monitoring of weight and dietary intake

*DXA* dual energy X-ray absorptiometry, *PHQ-9* Patient Health Questionnaire-9, *C-SSRS* Columbia Suicide Severity Rating Scale

Studies are needed to evaluate the benefits of consuming specific types of foods with second-generation AOMs. The literature, however, on large, rapid weight losses with very-low-calorie diets and bariatric surgery has shown the importance of prioritizing servings of lean protein to help preserve LBM [85]. Thus, to mitigate the potential loss of LBM with AOMs, we provisionally recommend that all patients consume a minimum of 60 g/day of high-quality protein [85], with a target of 0.8 g/day of protein per kilogram of body weight [86]. Higher amounts of protein may be appropriate with patients with higher BMIs who resemble individuals who undergo bariatric surgery [85]. Daily inclusion of a high-protein shake(s) may help meet these targets. We further recommend a diet that promotes cardiometabolic health, with an emphasis on increased fruits, vegetables, fiber, and other nutrient-rich foods, combined with decreased consumption of foods that are high in saturated fat and/or sugar [87]. Dietary strategies can be based on the patient’s comorbidities, as well as sociocultural preferences [88], and incorporate reduced-calorie versions of the US Dietary Guidelines [87], a Mediterranean-style diet [89], the DASH diet [90], or a variety of other approaches [6, 88]. MyPlate, developed by the US Dietary Guidelines (available at MyPlate.gov), provides patients a simple, accessible strategy for consuming a healthy dietary pattern [87].

Nutrient deficiencies are a common concern with marked energy restriction, as with very-low-calorie diets and bariatric surgery and could occur with some patients who achieve substantial weight loss with AOMs [91, 92]. Micronutrients of specific concern include vitamin B_12_, folate, thiamin, magnesium, potassium, calcium, the fat-soluble vitamins (A, D, E, K), iron, copper, zinc, and selenium [91, 92]. In the absence of official guidelines for micronutrient supplementation with energy-restricted diets [91], practitioners may wish to recommend a daily multivitamin for all patients taking AOMs and provide appropriate assessment and referral for nutritionally at-risk patients [92].

### Dietary Control of AOM-Related Side Effect

Dietary counseling with AOMs can also provide strategies to help mitigate common adverse effects of the medications, particularly nausea, diarrhea, vomiting, and constipation, which tend to be most prominent during dose escalation. GLP-1 agents do not interact with foods, but we suggest avoiding fatty, fried, greasy, and high-sugar foods for health reasons and to decrease GI side effects. Additional suggestions to decrease GI symptoms include consuming food slowly, having smaller meals, eating food that is light (and less spicy/acidic), avoiding eating too late at night, and maintaining hydration, with recommended fluid intakes of 2.2 L/day for women and 3 L/day for men. Patients should be informed of the signs and symptoms of dehydration (e.g., decreased urine, dizziness, dry mouth) and advised to keep non-caloric fluids with them and to drink slowly and frequently throughout the day if they experience nausea. Decreasing alcohol and caffeine can also help to avoid dehydration. This counseling, similar to that provided post-bariatric surgery patients, may help increase the tolerability of the medications and prevent treatment discontinuation.

## Physical Activity with Second-Generation AOMs

The STEP and SURMOUNT trials both found that weight loss was associated with participants’ reports of improved physical function [1••, 2••]. This is positive news, but it is not known whether this improvement was associated with participants adopting a more physically active lifestyle. As with dietary-intake findings, few data have been published from these trials on potential changes in participants’ physical activity or cardiorespiratory fitness occurring with weight loss, an absence also detected by a recent meta-analysis of the literature [93]. This underscores the need to quantify these outcomes and to examine the health benefits of coupling physical activity and improved cardiorespiratory fitness with weight loss achieved with AOMs. Patients and practitioners should be mindful that physical activity, independent of weight status, is associated with reduced risks of mortality, numerous cardiometabolic conditions, some forms of cancer, functional disabilities, and other complications [17]. Successful weight loss with AOMs may help patients enjoy physical activity for its recreational, social, and health benefits alone, without the need to use exercise to achieve negative energy balance, as it is with lifestyle modification. One patient remarked that he no longer experienced “the dread of having to exercise in order to lose weight” but instead enjoyed the greater physical activity that AOM-induced weight loss had afforded him.

### Increasing Physical Activity

We recommend that patients treated with second-generation AOMs seek to achieve a level of physical activity consistent with public health guidelines, with personalization of the activity plan based on the patient’s medical status and personal preferences [17]. These guidelines include the following:

- Progression to the equivalent of at least 5 day/week of 30 min of moderate-intensity aerobic physical activity (e.g., brisk walking). This progression can occur over a period of 3 to 6 months, the activity can be divided over multiple shorter episodes each day rather than need to complete all 30 min in one continuous period, and it can include activities other than walking that may be more enjoyable or more appropriate based on the patient’s physical capacity. This level of activity should be sustained to assist with weight loss maintenance and may need to increase to ≥ 60 min per day, particularly if patients decrease their AOM use. Patients can use a variety of devices (e.g., smart phones, watches, step counters) to track their activity.
- At least 2 days of muscle strengthening activities. This activity could be beneficial given the observed loss of lean mass with AOMs and may contribute to muscle strength that facilitates engagement in activities of daily living and a more physically active lifestyle. Muscle strengthening may be particularly important for older adults to reduce the risk of sarcopenia that contributes to reduced physical function.
- Balance training. This activity is also recommended for older adults and may be useful for younger individuals during weight loss to enhance their kinesthetic awareness and contribute to safe mobility and physical activity.

Given the significant energy deficit induced by the new AOMs, patients should be advised to gradually increase their physical activity, to not exercise to exhaustion, and to maintain adequate hydration. Practitioners also should counsel patients on the appropriate progression of physical activity based on the presence of comorbidities or other concerns.

### Effects of Strength Training

We agree with public health guidelines that recommend the inclusion of strength (resistance) training as part of physical activity [17]. However, there is currently limited evidence to demonstrate that adding such exercise to an AOM will prevent or minimize the reduction in LBM. For example, in response to a very-low-calorie diet (VLCD), which induced the loss of ~ 20% of baseline weight (~ 20 kg), the addition of structured exercise (i.e., randomization to aerobic, resistance, or aerobic plus resistance groups) did not preserve lean mass significantly better than the VLCD alone [94]. The same lack of benefit was observed in a similarly designed randomized study that produced a loss of ~ 15% with a 925-kcal/day meal-replacement diet [95]. More encouraging results were obtained in a VLCD study which induced a 16-kg weight loss. Tissue biopsy revealed muscle hypertrophy in participants who engaged in resistance training, compared with non-exercising controls, despite comparable weight losses and changes in body composition in the two groups [96]. In an observational analysis of Roux-en-Y gastric bypass patients (from a randomized trial), the greatest increase in walking was associated with the best preservation of skeletal muscle, measured at the thigh, and with less loss of total body mean mass [97]. When added to weight loss resulting from Roux-en-Y gastric bypass, walking also enhanced muscle quality [97]. These findings warrant further examination with weight loss induced by the new AOMs.

Future research can be guided, in part, by a study that examined the efficacy, for maintaining a 12% weight loss (achieved with a low-calorie diet run-in), of placebo, liraglutide 3.0 mg, exercise alone, or the combination of liraglutide plus exercise. At 1 year, the combination therapy was associated with significantly greater improvements than all other groups in body-fat percentage, cardiorespiratory fitness, and general heath perceptions [98]. These data provide eloquent testimony of the benefits of patients engaging in physical activity to improve their physical health and quality of life, not just their weight.

## Behavioral and Psychosocial Issues, Including Terminating AOMs

Many questions remain about the frequency and content of the behavioral (i.e., lifestyle) counseling required with the new AOMs. Based on current evidence, we recommend that brief diet and physical activity counseling be provided on the approximately monthly schedule used in the STEP trials, with two visits during the first month. More frequent meetings do not appear necessary to induce a 15–20% weight loss with the new AOMs, and some visits likely can be conducted by telephone or video-conferencing without loss of efficacy [31, 99]. Asynchronous, digitally delivered programs, which involve minimal person-to-person contact, also are likely to be explored [100]. In traditional office practice, RDs initially would appear to be best prepared to provide lifestyle counseling, but a variety of primary care professionals including physicians, nurse practitioners, nurses, psychologists, and medical assistants have provided such care following a structured behavioral protocol [12]. If not on staff, we encourage primary care practices to establish consulting relationships with RDs, certified clinical exercise physiologists, and psychologists for patients who need additional support in these respective areas.

### Selecting Weight Loss Goals and Related Outcomes

We begin all weight management efforts by first examining patients’ desire for treatment, their understanding of the nature and course of therapy they will receive, and the benefits, risks, and challenges that they can expect. This includes discussing how much weight they wish to lose and how they chose their particular number. Twenty years ago, lifestyle-treated participants typically reported desiring to lose 25% or more of their baseline weight [101], which was often followed by practitioners’ extolling the health benefits of a 5–10% reduction, the mean loss they could deliver at the time [8]. Losses of 15–25% now appear to be within reach with second-generation AOMs and will be particularly helpful in treating obesity-related complications such as obstructive sleep apnea, osteoarthritis, and non-alcoholic steatohepatitis (NASH), which require large reductions for optimal control [102]. In addition to enhancing their physical health, we ask patients to discuss other outcomes they hope to achieve with weight loss, including improvements in mobility, energy, and self-esteem, as well as engaging in new activities, such as learning to dance, play a new sport, or travel with family [12]. We encourage them to focus on their achievement—and enjoyment—of these functional goals rather than on reaching a number on the scale.

Obesity experts and health care professionals have much to learn about prescribing the new AOMs in clinical practice. The following hypothetical case illustrates the need for prudent clinical guidelines—beyond criteria provided by the FDA—which account for an individual’s age, sex, current health status (and history), medication use, and related factors. A 72-year old male with a BMI of 27.3 kg/m^2^ (weight of 190 lb, and height of 70 in) has elevated triglyceride levels. He has asked his doctor to prescribe semaglutide so that he can get down to his freshman-year weight (162 lb) for his 50th college reunion. He appears eligible for the medication but is it in the best interests of his health, given his age and risk of sarcopenia? With individuals such as these, practitioners may still wish to extoll the virtues of a more moderate 5–10% weight loss, even though larger reductions are possible. A larger weight loss may not always be a healthier weight loss [103]. In this case, the patient could achieve a therapeutic outcome with traditional lifestyle modification alone; an older, less robust AOM; a smaller dose of semaglutide (to limit weight loss); or with physical activity training alone to improve strength and conditioning. Such options may also be more appropriate for individuals who have obesity but are free of weight-related complications (i.e., metabolically healthy). Similarly, the benefits of more moderate weight loss, achieved at a low cost with traditional lifestyle modification, should not be overlooked in preventing type 2 diabetes [20] or in reducing mild elevations in blood pressure and other cardiometabolic risk factors [24].

### Self-Monitoring of Diet and Physical Activity

Additional questions concern the recommended frequency of patients’ monitoring their food and energy intake, the cornerstone of traditional lifestyle modification (and a more challenging task than tracking physical activity). Participants in most of the STEP and SURMOUNT trials had relaxed requirements for dietary self-monitoring and still lost approximately 15 to 20% of baseline weight, to which the lifestyle counseling program appeared to contribute only 2.5–3.0 percentage points (as suggested by the results for placebo-treated participants). Thus, frequent dietary self-monitoring does not appear to be necessary for successful weight loss with second-generation AOMs, although better data are needed to reach firm conclusions.

The frequency, however, of self-monitoring—and of lifestyle intervention contacts—needed to adopt a healthier dietary pattern may be substantially greater than that required to lose weight. Individuals, for example, who eat a diet of principally fast foods—or other highly processed items—likely will lose weight on AOMs simply by reducing the quantity of such foods consumed (resulting from decreased hunger and increased satiety), with little self-monitoring. By contrast, greater education, planning, self-monitoring, and lifestyle counseling likely would be required to shift from a diet of fast foods to a Mediterranean-style pattern, discussed earlier, with its potentially greater health benefits (beyond weight loss). Moreover, some patients likely will need more than monthly lifestyle counseling visits to consistently achieve the daily recommended intake of protein, an outcome not reported in any of the phase 3 trials. Regular self-monitoring also facilitates increased physical activity [8–12]. Thus, we recommend frequent monitoring of food intake and physical activity, as well as AOM adherence—perhaps the most critical behavior—during the first month to help patients identify their daily success with treatment adherence and needed behavior change. Provision of at least two lifestyle counseling visits the first month should help facilitate achievement of these goals. The intensity of self-monitoring—and of lifestyle intervention contacts—can be adjusted thereafter based on the patient’s specific goals for diet and activity change.

### Psychosocial Function

We believe that patients should be screened before treatment, as they were in phase 3 clinical trials, to determine that they are free of symptoms of current major depression and active suicidal ideation and behavior, as well as other significant psychopathology. These conditions can be assessed by interview, supported by screening instruments such as the Patient Health Questionnaire-9 (PHQ-9) [104] and the Columbia Suicide Severity Rating Scale [105]. In approving semaglutide, the FDA recommended that patients be monitored for the possible occurrence of these adverse events. This guidance was based on a history of such complications with some prior AOMs (e.g., rimonabant), although a warning was not provided for semaglutide per se [4]. We explain to patients why we ask about thoughts of possible self-harm, since some seem surprised, or even offended, by the questions.

The effects of semaglutide and tirzepatide on possible changes in mood and suicidal ideation (or behavior), as assessed in the STEP and SURMOUNT trials, have not been fully reported yet, as they were for liraglutide 3.0 mg, which had a generally favorable neuropsychiatric profile [106]. To date, no randomized controlled trials have reported consistently higher rates of any adverse psychiatric events in patients treated with semaglutide or tirzepatide compared with placebo [1••, 2••, 50, 51, 52••, 53••, 54–56]. However, at the time of this writing, the European Medicines Agency was examining post-marking surveillance data that included about 150 reports of possible cases of self-injury and suicidal thoughts in persons using GLP-1 receptor agonists (for type 2 diabetes or obesity) [107]. It is difficult with such data to determine whether the adverse events are potentially related to the medication’s use or are unrelated and would have occurred in its absence. Regardless of the possible causal relationship, immediate intervention is required with persons who report active suicidal ideation when it is characterized by a wish to die, an intent to act on the wish, and a specific plan to end one’s life. Practitioners unfamiliar with the assessment of suicide risk and how to respond to it can obtain further information by completing the on-lining training that accompanies the Columbia Suicide Severity Rating Scale (https://CSSRS.columbia.edu/training/training-options) [105].

We also screen for the presence of bulimia nervosa [108], which we believe is a contraindication to weight reduction and should prompt referral to an eating disorders specialist. We also check for a history of bulimia nervosa and anorexia nervosa, the latter which is rare in our patients seeking weight management. Further research, in collaboration with mental health professionals, is needed on the use of second-generation AOMs by patients with a history of either of these eating disorders to determine whether substantial weight loss triggers a recurrence of negative weight-related cognitions and behaviors and potentially of the full eating disorder. We would delay using semaglutide and tirzepatide with such individuals until appropriate guidance is available. Studies also are needed on the effects of second-generation AOMs on binge-eating disorder (BED), as well as on night eating syndrome, in persons with overweight and obesity. Participants with BED were excluded in some of the phase 3 studies discussed previously. However, this disorder was recently shown to improve in patients with overweight/obesity who were treated with naltrexone bupropion ER [109].

Psychosocial outcomes including quality of life (QOL) and mood generally improve with behavioral weight reduction, with larger losses typically associated with greater benefits [110, 111]. The STEP 1, 2, and 4 trials found greater improvements in weight- and physical health–related QOL at week 68 in patients treated with semaglutide compared with placebo [1••, 50, 52••]. Detailed findings concerning mood and other psychological outcomes await further reporting from the phase-3 trials, as described previously.

We expect that AOMs will produce improvements, on average, in many psychosocial outcomes. Nonetheless, we believe practitioners must be alert to the possibility of untoward psychosocial effects. Some individuals who achieve large weight losses, as with bariatric surgery, report unanticipated interpersonal challenges such as receiving unwelcomed comments about their shape and weight, being treated differently than when they weighed more (and felt ignored or invisible), renegotiating interpersonal relationships that centered around food, or terminating unhappy marriages [112]. A minority of patients, particularly those with a history of physical and/or sexual abuse, may subliminally experience excess weight as a protective factor and feel traumatically anxious or vulnerable after weight loss [113]. Reports in bariatric surgery patients of an elevated risk of suicidality, generally 3 or more years post-surgery, are rare but may be relevant to a small number of individuals who achieve substantial weight loss with AOMs [110]. As second-generation AOMs are increasingly used by the general population and potentially by individuals who have more severe depression or anxiety (or significant personality disorders) than study participants had, rates of psychiatric events could increase, as will be assessed by post-marketing surveillance by FDA and other regulatory bodies. We believe this possibility owes principally to the elevated current and lifetime rates of depression and anxiety in persons with obesity, particularly those with a BMI > 40 kg/m^2^, who often experience the greatest ill effects of weight-related stigmatization [99, 114, 115].

In cases in which significant psychosocial distress or dysfunction does not resolve with empathic support and reassurance from a primary care provider—and in which immediate intervention is not required, as with active suicidal ideation—we recommend that patients be referred to a mental health professional for further evaluation. Such referral may include a small number of individuals who wish to lose more weight after already having achieved a relatively low BMI (e.g., 20–22 kg/m^2^). We have observed that such individuals often do not experience improvements in body image or self-esteem, propelling them to seek further weight loss as a solution (a behavior also seen in individuals with anorexia and bulimia). Cognitive-behavioral treatment for body image is likely to be more effective [116].

### Long-Term Use of AOMs and Potential Discontinuation

Most patients likely will benefit from remaining on AOMs indefinitely to facilitate the maintenance of lost weight, as reviewed earlier [53••, 64]. We believe it is important to engage patients, as treatment progresses, in the discussion of long-term medication adherence, rather than stating at the outset, “you’ll need to take this medication for the rest of your life.” We often say “you can take the medication for as long as it’s helpful,” giving patients a sense of choice and control. Practitioners can clarify that medication will help patients lose weight in the first year and then, in ensuing years, support the equally important work of “keeping it off.” We also think it is important to acknowledge patients’ statements that “the medication’s not working as well—or any longer” when their weight loss plateaus or their subjective appetite control declines [47, 117]. Acknowledgement of disappointment or frustration can be joined with recognition that “the medication is still working to help you keep off 30 lb and control your diabetes.”

Several factors may lead patients to consider discontinuing AOMs including (1) concerns about their long-term safety, (2) patients’ beliefs that they should now be able to control their weight on their own (a potential form of self-stigmatization), and (3) the continued high costs of AOMs for weight-loss maintenance. Costs potentially could be reduced by less frequent dosing (e.g., every other week), as suggested with other medications [118] or by switching to an older, less expensive AOM (e.g., phentermine-topiramate, liraglutide), although this latter approach needs to be tested in a controlled trial to assess weight-loss maintenance following termination of semaglutide or tirzepatide. Some of our patients also have taken drug holidays for a month or more, with knowledge that they can return to the medication when needed, such as during the winter holidays. Research is urgently needed on cost-effective methods of facilitating weight-loss maintenance with AOMs.

Individuals who elect to discontinue AOMs should know that patients, on average, regain about two-thirds of lost weight in the ensuing year, a disheartening and daunting statistic [63••]. However, we have observed clinically that a small minority of patients maintain much of their weight loss after medication withdrawal. For 2 to 3 months before stopping a second-generation AOM, patients should be assisted in adopting a behavioral weight loss maintenance program, consistent with that practiced by participants in the National Weight Control Registry [32]. This could include enrolling in a structured behavioral program, as offered at academic medical centers or by some commercial providers. Better understanding of the effects of terminating AOMs on subjective and objective aspects of appetite and eating behavior could help patients address challenges in the initial weeks and months after drug discontinuation. Gradually reducing the medication dose, over 2–3 months—much as it was gradually introduced—could help ease discontinuation. The challenges to follow with long-term behavioral weight control are well known [30–35].

## Conclusions

Much like the discovery of highly effective medical therapies for hypertension, type 2 diabetes, and lipid disorders that transformed the management of these diseases, we believe that a new generation of nutrient stimulated hormone-based therapies has the potential to do the same for the treatment of obesity and its associated complications. Investigators and practitioners have much to learn about second-generation AOMs, including further understanding the mechanisms by which they consistently lower the point of body weight regulation by 15% or more [64]. Both semaglutide and tirzepatide, for example, appear to induce weight loss principally by reducing energy intake, but do they also attenuate reductions in resting and non-resting energy expenditure (i.e., metabolic adaptation) that thwart weight loss with diet and physical activity alone? As discussed throughout this paper, much also remains to be determined about how best to encourage patients to adopt healthier dietary patterns and physical activity routines, needed for optimal health, when such behaviors initially may not appear to be as important for weight loss with the new AOMs.

We look forward to the development of clinical practice guidelines, as well as standards of care, to ensure the best possible use of the new AOMs. Such guidelines, as successfully developed for the practice of metabolic and bariatric surgery [85, 110], must stress the importance of treating persons with overweight and obesity with respect, dignity, and compassion. This includes recognizing, assessing, and treating the many physical and psychosocial complications that some patients with obesity experience and which are not ameliorated by weight reduction alone. Multidisciplinary care is essential for such individuals.

Bold, persistent effort also will be required at a societal level to ensure that persons from socioeconomically disadvantaged populations, who disproportionately bear the burden of obesity and its health complications, have access to second-generation AOMs. This will include reducing the high costs of these medications [119] and assuring their coverage by public and private insurers [120]. Finally, our ability to treat obesity more effectively, on an individual level, does not diminish the dire need to ameliorate the obesogenic environment that continues to fuel this pandemic on a population level in the USA and other nations [121].

## Data Availability

No new original data were collected or produced in completing this article. The authors reviewed and synthesized selective literature (as referenced in the article) and then provided their recommendations for combining lifestyle modification with anti-obesity medications. We have no new data to share with other researchers.
